# Anatomical Burden of Prior Percutaneous Coronary Intervention and Long-Term Outcomes After Coronary Artery Bypass Grafting: An Analysis Spanning 2 Decades

**DOI:** 10.1093/icvts/ivaf237

**Published:** 2025-09-27

**Authors:** Go Yamashita, Jiro Sakai, Takumi Takauchi, Shun Otani, Shoya Nakano, Ryo Fujimoto, Atsushi Sugaya, Shingo Hirao, Tatsuhiko Komiya

**Affiliations:** Department of Cardiovascular Surgery, Kurashiki Central Hospital, Kurashiki, Okayama 710-8602, Japan; Department of Cardiovascular Surgery, Kurashiki Central Hospital, Kurashiki, Okayama 710-8602, Japan; Department of Cardiovascular Surgery, Kurashiki Central Hospital, Kurashiki, Okayama 710-8602, Japan; Department of Cardiovascular Surgery, Kurashiki Central Hospital, Kurashiki, Okayama 710-8602, Japan; Department of Cardiovascular Surgery, Kurashiki Central Hospital, Kurashiki, Okayama 710-8602, Japan; Department of Cardiovascular Surgery, Kurashiki Central Hospital, Kurashiki, Okayama 710-8602, Japan; Department of Cardiovascular Surgery, Kurashiki Central Hospital, Kurashiki, Okayama 710-8602, Japan; Department of Cardiovascular Surgery, Kurashiki Central Hospital, Kurashiki, Okayama 710-8602, Japan; Department of Cardiovascular Surgery, Kurashiki Central Hospital, Kurashiki, Okayama 710-8602, Japan

**Keywords:** coronary artery bypass grafting, prior percutaneous coronary intervention, coronary stents, myocardial infarction

## Abstract

**Objectives:**

This study aimed to determine whether the anatomical burden of prior percutaneous coronary intervention (PCI) influences long-term outcomes after coronary artery bypass grafting, beyond the impact of intervention presence alone.

**Methods:**

This retrospective study analysed consecutive patients undergoing coronary artery bypass grafting at a single institution between 2000 and 2024. The inclusion criteria comprised isolated, non-emergent surgery. Patient categorization was based on prior PCI-treated lesions: none, single, or multiple. The primary end-point was long-term overall survival. The secondary end-points included cardiac death, myocardial infarction, stroke, heart failure hospitalization, and repeat revascularization. Long-term outcomes were assessed using Kaplan-Meier analysis and Cox multivariable models, adjusting for 26 clinical factors.

**Results:**

Of 2442 patients, 1205 met the inclusion criteria (755 none, 227 single-lesion, 223 multiple-lesion intervention). Over a median follow-up of 12.0 (interquartile range, 11.3-12.9; maximum: 24.2) years, the multiple-lesion intervention group had higher rates of in-hospital acute kidney injury (34.1% vs 21.1% vs 24.2%, *P* = .003). Overall survival differed significantly between groups over the follow-up period (log-rank *P* = .004), with 15-year survival rates of 35.8%, 46.0%, and 48.0% for multiple-lesion, single-lesion, and no prior PCI groups, respectively. After adjustment, multiple-lesion intervention was associated with increased risks of cardiac death (adjusted subdistribution hazard ratio: 1.91), myocardial infarction (2.26), and repeat revascularization (1.92) compared with no prior intervention.

**Conclusions:**

Multiple-lesion PCI was associated with higher long-term risks of cardiac death, myocardial infarction, and repeat revascularization, while stroke risk was similar. Single-lesion PCI showed outcomes comparable to no prior PCI except for higher heart failure hospitalization. These findings require confirmation in larger, multicentre comparative studies to address residual confounding.

**Clinical registration number:**

4456.

## Graphical Abstract

**Figure:**
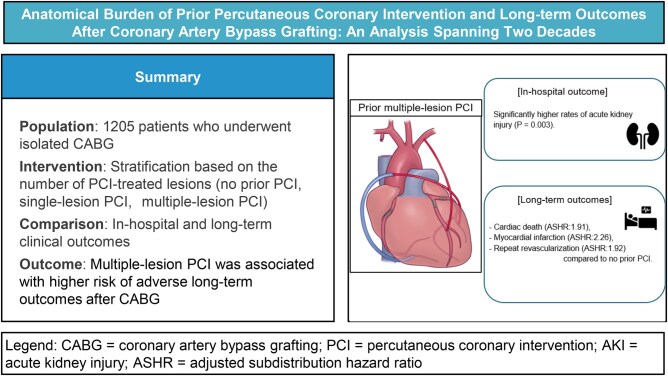

## INTRODUCTION

Coronary artery bypass grafting (CABG) remains the standard treatment for complex coronary artery disease, particularly in patients with diabetes and high anatomical complexity.^1^^,^^2^ However, the approach to coronary revascularization has evolved considerably, with more patients now undergoing CABG after percutaneous coronary intervention (PCI). A contemporary analysis of the Society of Thoracic Surgeons Database revealed that the proportion of patients with prior PCI who subsequently underwent CABG increased from 20.0% in 2008 to 25.0% in 2018.^3^^,^^4^ The impact of prior PCI on CABG remains debatable.^5–9^ Meta-analyses have suggested that previous PCI may negatively impact early postoperative outcomes, including higher in-hospital and 30-day mortality.^6–8^ However, most studies have primarily examined the presence or frequency of prior PCI,^9^ potentially overlooking the anatomical complexity of PCI-treated lesions. This issue is particularly relevant as cardiac surgeons frequently encounter technical challenges when constructing bypass grafts in vessels with multiple stents, potentially affecting short- and long-term outcomes. However, the relationship between the number of PCI-treated lesions and subsequent CABG outcomes remains unclear. Despite established associations between prior PCI and CABG outcomes, the specific impact of anatomical PCI burden on long-term clinical events after CABG has not been systematically evaluated.

Therefore, we evaluated how the number of prior PCI-treated lesions, rather than the mere presence or frequency of PCI, influences long-term clinical outcomes following CABG. To investigate this relationship, we analysed over 2 decades of consecutive CABG procedures performed at a single institution.

## PATIENTS AND METHODS

### Ethical statement

This retrospective, observational, single-centre study was approved by the Institutional Review Board of Kurashiki Central Hospital (Approval No. 4456; Approval Date: August 6, 2024). The requirement for informed consent was waived due to the retrospective nature of the study. Any collection and storage of data or biological material from research participants for multiple and indefinite use was conducted in accordance with the WMA Declaration of Taipei. The research ethics committee approved the establishment and ongoing use of such databases and biobanks.

### Study design and population

We retrospectively analysed data from consecutive adult patients (aged ≥ 18 years) who underwent CABG at our institution between January 2000 and January 2024 (*n* = 2442). The exclusion criteria were: (1) concomitant cardiac procedures, including valve surgery or aortic procedures; (2) emergent operations requiring immediate surgical intervention. No patients were excluded based on sex, comorbidities, or the number of bypass grafts. After applying these criteria, only patients who underwent isolated CABG were included in the final analysis.

### Definition of prior PCI lesions and patient classification

The coronary artery system was divided into 3 main regions: left anterior descending artery (LAD), left circumflex artery (LCx), and right coronary artery (RCA). Patients were categorized into 3 groups based on the location of prior PCI-treated lesions: no prior PCI (patients with no history of PCI), single-lesion PCI (patients with PCI in 1 coronary region), and multiple-lesion PCI (PCI in 2 or more regions). Prior stent implantation in the left main trunk was considered as 2-region involvement and classified into the multiple-lesion PCI group. The frequency of PCI procedures did not influence group classification. For instance, patients who underwent multiple PCIs in the same region (eg, 2 procedures in the LAD) were classified into the single-lesion PCI group.

### Definitions for baseline characteristics and outcome measures

The primary end-point was long-term overall survival (OS). The secondary end-points included cardiac death, myocardial infarction (MI),^10^ stroke, heart failure hospitalization, and repeat revascularization. Detailed definitions of all baseline characteristics and outcome measures are provided (see **Supplementary Method**). In-hospital outcomes, including acute kidney injury (AKI) (assessed using established criteria^11^), bleeding complications, mechanical support requirements, and mortality, were comprehensively evaluated to assess perioperative safety across groups. Patient characteristics and operative data were obtained from medical records. Follow-up data were collected through outpatient clinic chart reviews, telephone interviews, and postal questionnaires for patients who did not attend the outpatient clinic. Data collection was completed on March 31, 2024. Given our annual follow-up protocol, patients who had clinic visits or were contacted within 12 months of this date were considered to have complete follow-up. Follow-up completeness was assessed using the percentage method, calculated as the proportion of patients with known vital status within 12 months of study end divided by the total number of enrolled patients.

### Surgical procedures

The surgical technique has been previously described.^9^ In the early 2000s, while the internal thoracic artery remained the primary conduit, the right gastroepiploic artery (RGEA) and radial artery (RA) were frequently incorporated as additional arterial conduits to facilitate an aorta-no-touch technique. The introduction of a harmonic scalpel (Ethicon Endo-Surgery Inc.) significantly improved internal thoracic artery harvesting,^12^ leading to increased adoption of bilateral internal thoracic artery grafting. Recently, there has been a shift towards incorporating no-touch saphenous vein grafts (NT-SVG) into our revascularization strategy^13^ (see **Figure S1**).

### Statistical analysis

Continuous variables are presented as mean (standard deviation) or median (interquartile range [IQR]) depending on normality, as assessed by Shapiro-Wilk test. Variables among groups were compared using 1-way analysis of variance or Kruskal-Wallis test, as appropriate.

Categorical variables are presented as counts and percentages and were compared using chi-square test or Fisher’s exact test. When significant group differences were found, post hoc pairwise comparisons used Fisher’s exact test with Holm’s correction. Trend analyses were performed to assess dose-response relationships across PCI burden categories. Cochrane-Armitage trend tests were used for binary outcomes and Jonckheere-Terpstra tests for continuous outcomes. Median follow-up time was calculated using the reverse Kaplan-Meier method. Long-term clinical outcomes, including OS, were estimated using Kaplan-Meier analysis. Differences were assessed by the log-rank test. For cardiac death, the cumulative incidence function was estimated with non-cardiac death as a competing risk. For other postoperative outcomes (MI, stroke, heart failure hospitalization, and repeat revascularization), cumulative incidence functions were estimated considering death as a competing risk. Differences between groups were compared using Gray’s test. The effects of multiple-lesion and single-lesion PCI, relative to no prior PCI, were estimated using Cox proportional hazards models for OS and Fine-Gray competing risk models for cause-specific outcomes. Adjusted hazard ratios (HRs) with 95% confidence intervals (CIs) and subdistribution HRs (sHRs) with 95% CIs were calculated using multivariable models incorporating 26 clinically relevant covariates (**Tables 1**  **and**  **2**), selected based on clinical importance, prior prognostic evidence,^14^ and baseline differences (*P* < .05). Missing data were assessed for all variables. Complete case analysis was performed for the multivariable models, as missing data were <1% for all baseline covariates. No imputation was performed, given the minimal missing data and assumption of missing completely at random mechanism. Patients were censored at death, last known contact, or March 31, 2024, whichever occurred first. All statistical analyses were performed using R software version 4.4.1 (The R Foundation for Statistical Computing, Vienna, Austria), with significance set at 2-sided *P* < .05.

**Table 1.** Baseline Characteristics

Characteristics	No PCI	Single-lesion PCI	Multiple-lesion PCI	*P*-value	*P* for trend
	*N* = 755	*N *= 227	*N* = 223		
Age, years, (SD)	67.4 (10.8)	66.7 (9.9)	67.6 (8.9)	.615	.307
Age ≥ 75^a^	192 (25.4)	56 (24.7)	49 (22.0)	.590	.312
Male^a^	595 (78.8)	181 (79.7)	176 (78.9)	.963	.907
BMI ≥ 25.0 kg/m²^a^	252 (33.4)	80 (35.2)	83 (37.2)	.546	.272
Hypertension^a^	589 (78.0)	189 (83.3)	195 (87.4)	.004	<.001
Hyperlipidaemia^a^	497 (65.8)	146 (64.3)	156 (70.0)	.404	.362
Current smoker^a^	120 (15.9)	36 (15.9)	20 (9.0)	.025	.021
Diabetes^a^	424 (56.2)	129 (56.8)	147 (65.9)	.031	.017
eGFR <45, without dialysis^a^	123 (16.3)	46 (20.3)	42 (18.8)	.313	.241
Dialysis^a^	60 (7.9)	20 (8.8)	43 (19.3)	<.001	<.001
Previous stroke^a^	122 (16.2)	33 (14.5)	45 (20.2)	.247	.265
Atrial fibrillation^a^	42 (5.6)	10 (4.4)	12 (5.4)	.838	.775
Old myocardial infarction^a^	223 (29.5)	139 (61.2)	130 (58.3)	<.001	<.001
Peripheral vascular disease^a^	134 (17.7)	40 (17.6)	45 (20.2)	.682	.465
Chronic obstructive pulmonary disease^a^	37 (4.9)	9 (4.0)	11 (4.9)	.832	.885
Anaemia (haemoglobin <11.0 g/dL)^a^	141 (18.7)	53 (23.3)	51 (22.9)	.179	.098
Thrombocytopaenia (platelets <100 × 109/L)^a^	21 (2.8)	1 (0.4)	2 (0.9)	.037	.027
LVEF ≤40%^a^	115 (15.2)	36 (15.9)	29 (13.0)	.651	.502
Bare metal stent	0	158 (69.6)	144 (64.6)	<.001	<.001
First-generation drug-eluting stent	0	31 (13.7)	80 (35.9)	<.001	<.001
Second-generation and newer drug-eluting stents	0	43 (18.9)	53 (23.8)	<.001	<.001
Drug-coated balloon	0	3 (1.3)	15 (6.7)	<.001	<.001

Continuous variables are expressed as mean (SD, standard deviation). Categorical variables are expressed as numbers (percentages).

a Risk-adjusted variables selected for Cox proportional hazards models.

Abbreviations: eGFR: estimated glomerular filtration rate; LVEF: left ventricular ejection fraction.

**Table 2.** Surgical Characteristics and Medication at Discharge

Characteristics	No PCI	Single-lesion PCI	Multiple-lesion PCI	*P*-value	*P* for trend
	*N* = 755	*N* = 227	*N* = 223		
Surgical characteristics					
Off-pump^a^	668 (88.5)	196 (86.3)	192 (86.1)	.515	.276
Number of anastomoses (SD)	3.31 (0.96)	3.31 (0.99)	3.23 (0.85)	.496	.313
Internal thoracic artery use^a^	752 (99.6)	222 (97.8)	221 (99.1)	.031	.167
Target of chronic total occlusion^a^	288 (38.1)	67 (29.5)	62 (27.8)	.003	.001
Endarterectomy^a^	14 (1.9)	8 (3.5)	5 (2.2)	.329	.469
Total arterial revascularization	255 (33.8)	79 (34.8)	66 (29.6)	.443	.334
Medication at discharge					
Antiplatelet therapy	744 (98.5)	222 (97.8)	215 (96.4)	.131	.046
Dual antiplatelet therapy	230 (30.5)	78 (34.4)	84 (37.7)	.106	.034
Aspirin^a^	721 (95.5)	214 (94.3)	198 (88.8)	.001	<.001
Clopidogrel^a^	163 (21.6)	52 (22.9)	67 (30.0)	.032	.014
Statins^a^	354 (46.9)	100 (44.1)	110 (49.3)	.532	.713
Beta-blockers^a^	323 (42.8)	82 (36.1)	94 (42.2)	.197	.517
ACE-I/ARB^a^	294 (38.9)	102 (44.9)	81 (36.3)	.147	.857

Continuous variables are expressed as mean (SD, standard deviation) or median (interquartile range). Categorical variables are expressed as numbers (percentages).

a Risk-adjusted variables selected for Cox proportional hazards models.

Abbreviations: ACE-I: angiotensin-converting enzyme inhibitor; ARB: angiotensin II receptor blocker.

## RESULTS

### Baseline characteristics

**Figure 1.**
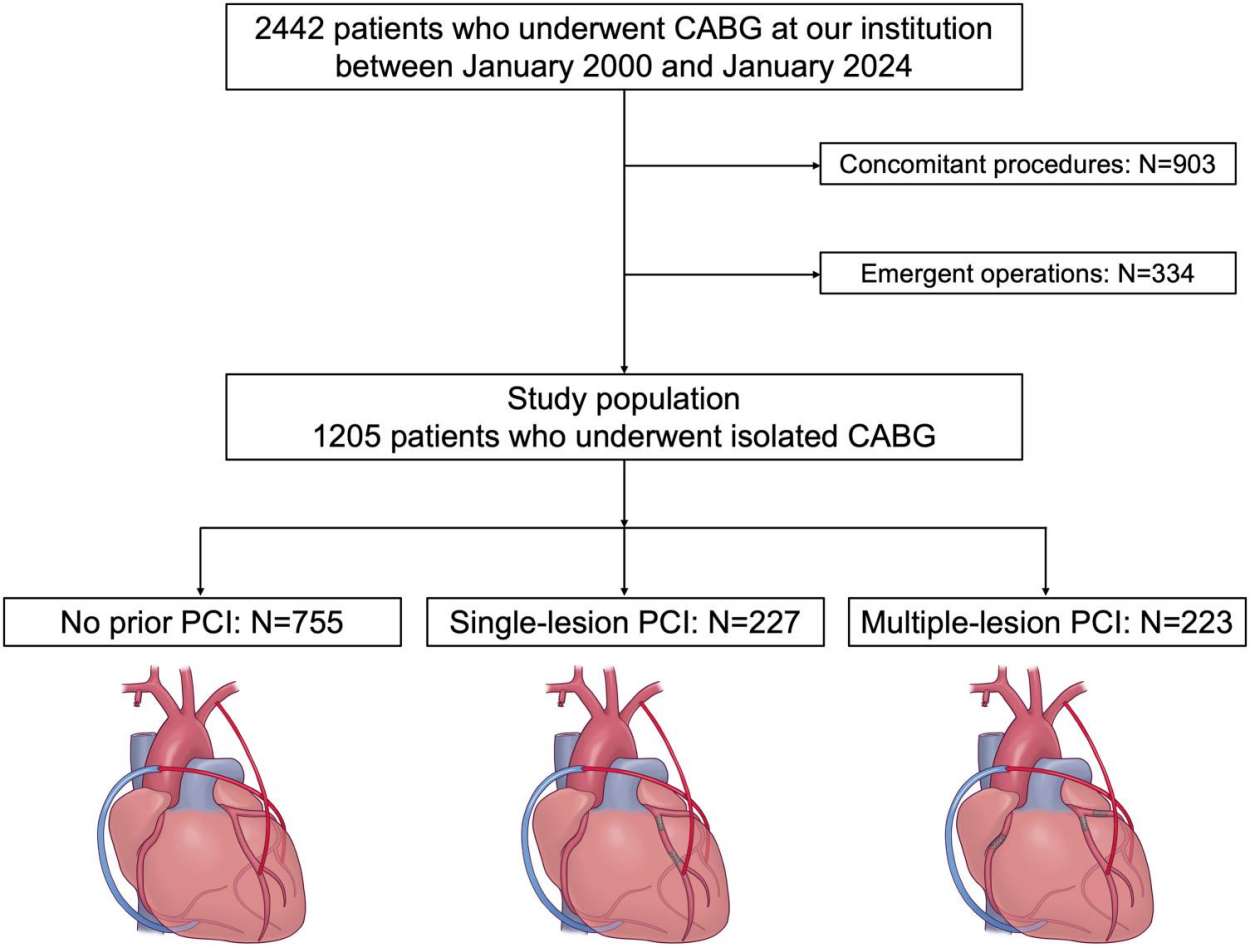
Flow Diagram Illustrating Patient Selection and Study Groups Classification. Abbreviations: CABG: coronary artery bypass grafting; PCI: percutaneous coronary intervention

The final study cohort comprised 1205 patients who underwent isolated CABG, stratified into 3 groups: no prior PCI (*n* = 755), single-lesion PCI (*n* = 227), and multiple-lesion PCI (*n* = 223) (**Figure 1**). From 2000 to 2024, the annual proportion of patients with prior PCI ranged from 17.6% to 52.2% (**Figure S2**). The prevalence of hypertension, eGFR, dialysis, and prior MI differed among groups, with prior MI being markedly higher in patients with previous PCI (no PCI: 29.5%, single-lesion: 61.2%, multiple-lesion: 58.3%; *P* < .001), suggesting more extensive coronary disease in these patients. However, left ventricular function was similar across groups, with comparable left ventricular ejection fraction (LVEF) rates ≤40% (15.2%, 15.9%, and 13.0%, for no prior PCI, single-lesion PCI, and multiple-lesion PCI, respectively; *P* = .651). Bare metal stents (BMS) were most commonly implanted (25.1%), followed by first-generation drug-eluting stents (9.2%). Off-pump CABG was performed in 87.6% of cases. The number of arterial grafts, targets of chronic total occlusion, and intraoperative blood transfusion varied. Most patients were prescribed antiplatelet therapy, with aspirin being the most common (94.0%), while clopidogrel use varied among groups. Significantly increasing trends for hypertension (*P* < .001), diabetes (*P* = .017), and dialysis (*P* < .001) were noted across PCI burden categories (**Tables 1**  **and**  **2** and **Table S1**).

### In-hospital clinical outcomes

The incidence of AKI differed significantly across groups (no prior PCI, 24.2%; single-lesion PCI, 21.1%; multiple-lesion PCI, 34.1%; *P* = .003, *P* for trend = .016). Holm’s post hoc test showed that the multiple-lesion PCI group had a higher incidence than both the single-lesion PCI (*P* = .007) and no prior PCI groups (*P* = .009), while no significant difference was found between the no prior PCI and single-lesion PCI groups (*P* = .373). In-hospital mortality was similar across all groups (*P* = .465). Other complications—re-exploration for bleeding, mechanical circulatory support, gastrointestinal bleeding, new-onset stroke, dialysis, deep wound infection, and prolonged ventilation—did not differ significantly (**Table 3**). The distribution of stented territories differed between groups (**Table S2**). In the single-lesion PCI group, RCA was most frequently stented (62.1%), while the multiple-lesion PCI group showed high rates of stenting across all territories, with particularly high LAD involvement (85.7%). Early graft patency rates are shown in **Table S3**.

**Table 3.** In-Hospital Clinical Outcomes

Outcome	No prior PCI *N* = 755	Single-lesion PCI *N* = 227	Multiple-lesion PCI *N* = 223	*P*-value	*P* for trend
Re-exploration for bleeding	15 (2.0)	6 (2.6)	10 (4.5)	.117	.044
IABP-support	20 (2.6)	6 (2.6)	7 (3.1)	.921	.724
ECMO-support	2 (0.3)	2 (0.9)	3 (1.3)	.141	.048
Gastrointestinal bleeding	4 (0.5)	1 (0.4)	4 (1.8)	.131	.090
New-onset stroke	16 (2.1)	6 (2.6)	9 (4.0)	.282	.121
New-onset dialysis	6 (0.8)	1 (0.4)	1 (0.4)	.769	.507
Deep wound infection	13 (1.7)	5 (2.2)	7 (3.1)	.422	.195
Prolonged ventilation	50 (6.6)	18 (7.9)	25 (11.2)	.078	.027
Acute kidney injury	183 (24.2)	48 (21.1)	76 (34.1)	.003	.016
In-hospital death	11 (1.5)	4 (1.8)	6 (2.7)	.465	.231

Categorical variables are expressed as numbers (percentages).

Abbreviations: ECMO: extracorporeal membrane oxygenation; IABP: intra-aortic balloon pump.

### Long-term clinical outcomes

**Figure 2.**
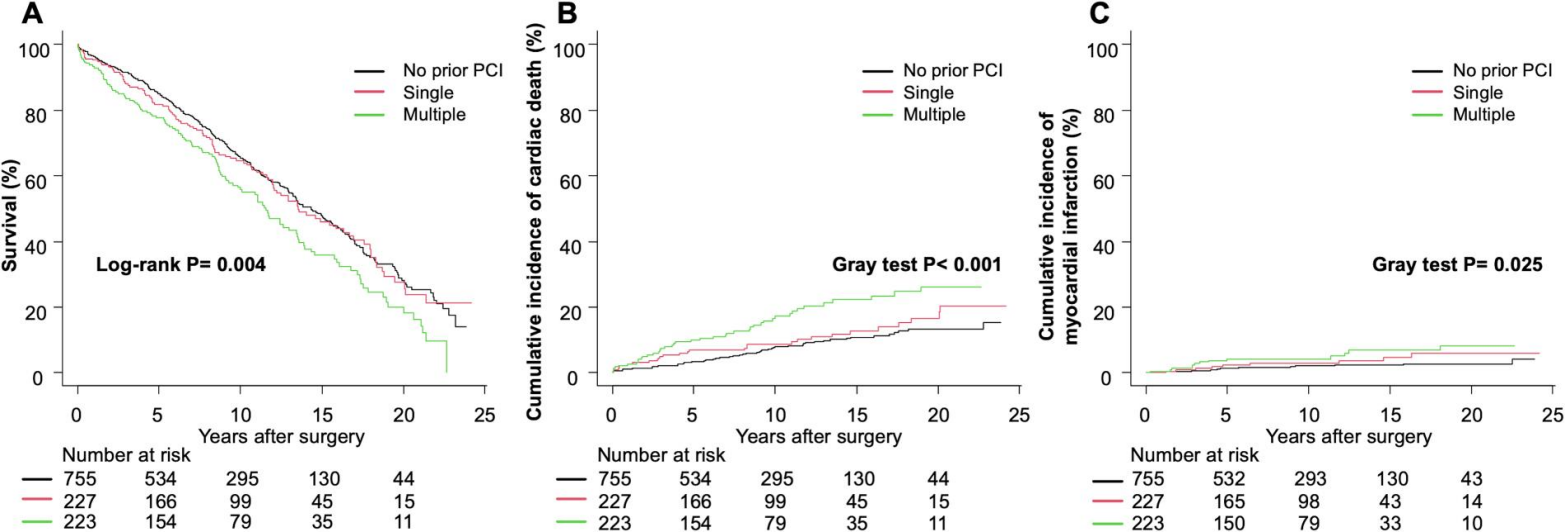
Clinical Outcomes According to Prior PCI Status. (A) Kaplan-Meier curves for overall survival. (B) Cumulative incidence curves for cardiac death with non-cardiac death as a competing risk. (C) Cumulative incidence curves for myocardial infarction with all-cause death as a competing risk. Abbreviation: PCI: percutaneous coronary intervention

**Figure 3.**
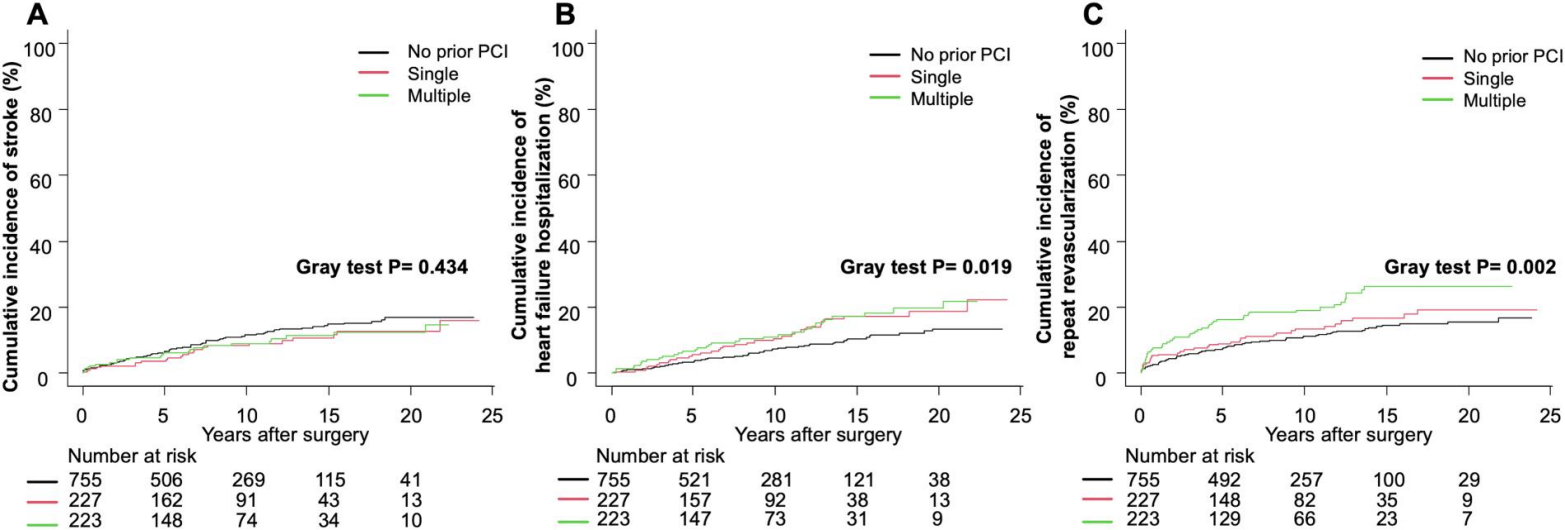
Clinical Outcomes According to Prior PCI Status (Continued). (A) Cumulative incidence curves for stroke with all-cause death as a competing risk. (B) Cumulative incidence curves for heart failure hospitalization with all-cause death as a competing risk. (C) Cumulative incidence curves for repeat revascularization with all-cause death as a competing risk. Abbreviation: PCI: percutaneous coronary intervention

The complete follow-up rate calculated per the percentage method was 92.4%. The median follow-up time for the entire cohort, calculated using the reverse Kaplan-Meier method, was 12.0 years (IQR: 11.3-12.9), with a maximum of 24.2 years. Median follow-up times were 11.3 years (10.6-12.5), 14.0 years (11.3-15.8), and 12.1 years (10.9-15.5) for the no prior PCI, single-lesion, and multiple-lesion PCI groups, respectively. Analysis of long-term outcomes revealed several key findings. OS differed significantly across groups over the entire follow-up period (log-rank *P* = .004; **Figure 2A**). At 15 years, the observed survival rates were 35.8%, 46.0%, and 48.0% for the multiple-lesion, single-lesion, and no prior PCI groups, respectively. For OS, analysed using Cox proportional hazards models, the risk of all-cause death in the multiple-lesion PCI group was numerically higher but not statistically significant (adjusted HR: 1.21; 95% CI: 0.97-1.52; *P* = .096). Competing risk analyses showed significant differences between groups for cardiac death (Gray’s test, *P* < .001; **Figure 2B**) and repeat revascularization (Gray’s test, *P* = .002; **Figure 3C**), with borderline significance for MI (Gray’s test, *P* = .025; **Figure 2C**) and heart failure hospitalization (Gray’s test, *P* = .019; **Figure 3B**). Stroke outcomes did not differ significantly (Gray’s test, *P* = .434; **Figure 3A**). After adjusting for confounders using Fine-Gray competing risk models (**Table 4**), the multiple-lesion PCI group showed higher risks of cardiac death (adjusted sHR: 1.91; 95% CI: 1.25-2.93; *P* = .003), MI (adjusted sHR: 2.26; 95% CI: 1.06-4.82; *P* = .035), and repeat revascularization (adjusted sHR: 1.92; 95% CI: 1.34-2.76; *P* < .001) compared with the no prior PCI group. For heart failure hospitalization, the multiple-lesion PCI group showed a trend towards increased risk (adjusted sHR: 1.54; 95% CI: 0.98-2.42; *P* = .059), while the single-lesion PCI group had significantly higher risk (adjusted sHR: 1.61; 95% CI: 1.01-2.56; *P* = .042). For stroke, no significant differences were observed among the 3 groups (adjusted sHR for multiple-lesion: 0.86; 95% CI: 0.53-1.38; *P* = .530).

**Table 4.** Long-Term Clinical Outcomes Based on the Number of Prior PCI Lesions

Outcome	Group	Crude HR (95% CI)	*P*-value	Adjusted HR (95% CI)	*P*-value
Survival	No prior PCI	Reference	–	Reference	–
	Single-lesion PCI	1.05 (0.85-1.30)	.662	0.96 (0.76-1.21)	.719
	Multiple-lesion PCI	1.42 (1.15-1.74)	<.001	1.21 (0.97-1.52)	.096
Cardiac death	No prior PCI	Reference	–	Reference	–
	Single-lesion PCI	0.97 (0.62-1.52)	.900	1.15 (0.68-1.96)	.600
	Multiple-lesion PCI	2.16 (1.51-3.08)	<.001	1.91 (1.25-2.93)	.003
Myocardial infarction	No prior PCI	Reference	–	Reference	–
	Single-lesion PCI	1.26 (0.58-2.74)	.560	1.97 (0.79-4.90)	.140
	Multiple-lesion PCI	2.20 (1.14-4.23)	.019	2.26 (1.06-4.82)	.035
Stroke	No prior PCI	Reference	–	Reference	–
	Single-lesion PCI	0.70 (0.43-1.15)	.160	0.62 (0.37-1.04)	.069
	Multiple-lesion PCI	0.97 (0.64-1.48)	.900	0.86 (0.53-1.38)	.530
Heart failure hospitalization	No prior PCI	Reference	–	Reference	–
	Single-lesion PCI	1.55 (1.04-2.32)	.034	1.61 (1.01-2.56)	.042
	Multiple-lesion PCI	1.34 (0.90-2.00)	.140	1.54 (0.98-2.42)	.059
Repeat revascularization	No prior PCI	Reference	–	Reference	–
	Single-lesion PCI	1.07 (0.72-1.59)	.720	1.35 (0.88-2.07)	.170
	Multiple-lesion PCI	1.70 (1.23-2.36)	.001	1.92 (1.34-2.76)	<.001

For overall survival: hazard ratios from Cox proportional hazards models. For cardiac death, myocardial infarction, stroke, heart failure hospitalization, and repeat revascularization: subdistribution hazard ratios from Fine-Gray competing risk models with all-cause death as a competing risk (non-cardiac death for cardiac death outcome). All models were adjusted for 26 baseline clinical variables.

Abbreviations: CI: confidence interval; HR: hazard ratio; PCI: percutaneous coronary intervention.

During follow-up, 36 patients experienced MI (detailed angiographic findings are provided in **Table S4**).

## DISCUSSION

The present study highlights several key findings regarding the impact of prior PCI treatment on subsequent CABG outcomes. First, patients who underwent multiple-lesion PCI had significantly higher rates of AKI during the immediate postoperative period than those with no prior PCI or single-lesion PCI. Second, multiple-lesion PCI was associated with worse long-term outcomes, including increased risk of cardiac death, MI, and repeat revascularization. Third, patients who underwent single-lesion PCI showed outcomes comparable to those without prior PCI for most end-points, except for heart failure hospitalization. These findings build upon previous studies that primarily focused on the presence or frequency of prior PCI procedures. While meta-analyses have suggested adverse effects of prior PCI on short-term outcomes after CABG,^6–8^ our study demonstrates that the anatomical burden of PCI-treated lesions, rather than the mere presence of prior PCI, may be a more relevant predictor of long-term outcomes. The higher incidence of postoperative AKI in the multiple-lesion PCI group warrants further investigation. This finding may be attributed to cumulative contrast exposure and higher prevalence of baseline renal dysfunction in this group. These observations underscore the need for meticulous perioperative renal management in patients with extensive PCI history.

Post-CABG MI analysis revealed 41.7% occurred in previously stented lesions with failed grafts, highlighting revascularization challenges. Technical difficulties include altered vessel architecture,^15^ compromised distal runoff,^6^ and limited anastomotic sites.^5^^,^^9^ The generally comparable outcomes between the single-lesion PCI and no prior PCI groups (except for heart failure hospitalization) suggest that limited PCI exposure may not significantly compromise surgical revascularization in most aspects, provided that appropriate patient selection and techniques are used. This has important implications for the “heart team” approach to revascularization, indicating that initial PCI of a single lesion may be reasonable for selected patients who may eventually undergo CABG.^14^

The extent of epicardial coronary artery disease has been identified as a strong predictor of perioperative MI following CABG.^16^ In our study, patients with multiple-lesion PCI likely had more extensive and diffuse coronary disease, which may have contributed to their higher perioperative complication rates and worse long-term outcomes. Although we did not systematically measure perioperative MI, the elevated incidence of AKI in the multiple-lesion PCI group may reflect their overall higher perioperative risk profile, as major perioperative complications often cluster in high-risk patients.

Our findings have significant implications for clinical revascularization strategies. The number of PCI-treated lesions has emerged as a crucial factor in risk assessment for subsequent CABG, beyond the traditional consideration of prior PCI. Patients with a multiple-lesion PCI require tailored perioperative strategies, particularly renal function monitoring, given their higher risk of AKI. Additionally, the technical challenges observed, especially the high rate of graft failure in previously stented vessels, underscore the importance of careful conduit selection and precise anastomotic site placement. The increased risk of adverse cardiac events in these patients highlights the need for a more rigorous post-CABG surveillance.

These observations also carry broader implications for the heart team’s decision-making. When determining initial treatment strategies for complex coronary artery disease, the potential impact of extensive stenting on future surgical options should be carefully weighed. This is particularly relevant given our finding that single-lesion PCI did not significantly compromise surgical outcomes, suggesting that limited PCI remains a viable option for patients who may eventually require CABG. This knowledge should inform revascularization planning, especially for younger patients with longer life expectancy. Additionally, our study reflects the historical context of coronary intervention, where BMS predominated over DES. However, the evolving stent landscape limits our ability to draw firm conclusions regarding newer-generation DES. Further studies are needed to evaluate the impact of current-generation stents on subsequent CABG outcomes.

This study has limitations. First, as a retrospective, single-centre study, its findings may have limited generalizability. Second, although multivariate adjustments were performed, unmeasured confounders may have influenced the results. Third, our definition of PCI burden based on the number of treated territories does not capture other important aspects, such as the total stent length or number of stents per vessel, which were not available in our database. These metrics might provide additional insights into the relationship between PCI complexity and CABG outcomes. Fourth, standardized risk scores, such as EuroSCORE or STS score, were not available due to the extended study period and evolving score algorithms. However, our multivariable models incorporated 26 clinical variables that encompass most risk factors included in these standardized scores. Finally, we did not collect data on post-infarction left ventricular aneurysm, which could influence surgical outcomes, particularly given the higher prevalence of prior MI in the PCI groups. While we adjusted for overall left ventricular function (LVEF ≤40%), this does not capture the specific impact of ventricular aneurysm.

## CONCLUSION

Over 2 decades, multiple-lesion PCI was associated with higher risks of cardiac death, MI, and repeat revascularization after CABG, while stroke did not differ materially among groups. Single-lesion PCI yielded outcomes broadly comparable to no prior PCI, aside from a higher risk of heart failure hospitalization; in contrast, multiple-lesion PCI showed only a trend towards increased heart failure hospitalization. Although our multivariable models adjusted for 26 covariates, unmeasured confounding remains possible. Accordingly, the anatomical extent of prior PCI should be considered in revascularization planning for surgical candidates, and confirmation in larger, multicentre cohorts with granular PCI metrics and prospective comparative designs is warranted before drawing definitive conclusions.

## Supplementary Material

ivaf237_Supplementary_Data

## Data Availability

The data underlying this article will be shared on reasonable request to the corresponding author.
