# Supplementary material for: Anatomical Burden of Prior Percutaneous Coronary Intervention and Long-Term Outcomes After Coronary Artery Bypass Grafting: An Analysis Spanning 2 Decades
Source: Interdiscip Cardiovasc Thorac Surg. 2025 Sep 27;40(10):ivaf237. doi: 10.1093/icvts/ivaf237 (PMC12516811; doi:10.1093/icvts/ivaf237)
Supplement: ivaf237_Supplementary_Data [file ivaf237_supplementary_data.zip › Supplemental_File_Sep9_2025.docx]

**Supplemental Material**

**Supplemental Method**

**Detailed definitions of baseline characteristics and outcome measures**

Baseline characteristics were defined as follows: hypertension, hyperlipidemia, current smoking, prior stroke, old myocardial infarction (MI), and peripheral vascular disease were considered present if documented in hospital records. Diabetes was defined by treatment with oral hypoglycemic agents or insulin, a previous clinical diagnosis, or a glycated hemoglobin level ≥6.5%. Chronic obstructive pulmonary disease was defined as a documented diagnosis or a forced expiratory volume in one second/forced vital capacity ratio <70% on spirometry. The primary endpoint was long-term overall survival (OS). Secondary endpoints included cardiac death, defined as death due to MI, heart failure, or sudden cardiac death; MI, defined according to the Academic Research Consortium criteria (10); stroke, defined as ischemic or hemorrhagic events with neurological symptoms lasting more than 24 hours; heart failure hospitalization, defined as admission requiring intravenous therapy or inotropic support; and repeat revascularization, which included both PCI and CABG. Acute kidney injury was defined according to the Kidney Disease: Improving Global Outcomes criteria (11).

**Supplemental Table**

| **Supplemental Table 1: Detailed baseline and surgical characteristics** | | | | | |  | |  |
| --- | --- | --- | --- | --- | --- | --- | --- | --- |
| **Characteristics ​** | **Overall** | **No PCI** | **Single-lesion PCI** | **Multiple-lesion PCI** | **P-value ​** | | **P for trend** | |
|  | N = 1205 | N = 755 | N = 227 | N = 223 |  |  |  |  |
| **(A) Baseline characteristics** |  |  |  |  |  | |  | |
| Age, years, (SD) | 67.3 (10.3) | 67.4 (10.8) | 66.7 (9.9) | 67.6 (8.9) | 0.615 | | 0.307 | |
| Age ≥ 75* | 297 (24.6) ​ | 192 (25.4) | 56 (24.7) | 49 (22.0) | 0.590 | | 0.312 | |
| Male* | 952 (79.0) | 595 (78.8) | 181 (79.7) | 176 (78.9) | 0.963 | | 0.907 | |
| Body mass index, kg/m², (SD) | 23.8 (3.5)​ | 23.7 (3.4) | 23.8 (3.6) | 24.3 (3.9) | 0.083 | | 0.101 | |
| BMI ≥ 25.0* | 415 (34.4) ​ | 252 (33.4) | 80 (35.2) | 83 (37.2) | 0.546 | | 0.272 | |
| Body surface area, m², (SD) | 1.66 (0.18)​ | 1.66 (0.17) | 1.66 (0.18) | 1.68 (0.18) | 0.309 | | 0.431 | |
| Hypertension* | 973 (80.7) ​ | 589 (78.0) | 189 (83.3) | 195 (87.4) | 0.004 | | <0.001 | |
| Hyperlipidemia* | 799 (66.3) ​ | 497 (65.8) | 146 (64.3) | 156 (70.0) | 0.404 | | 0.362 | |
| Current smoker​* | 176 (14.6) ​ | 120 (15.9) | 36 (15.9) | 20 (9.0) | 0.025 | | 0.021 | |
| Diabetes​* | 700 (58.1) ​ | 424 (56.2) | 129 (56.8) | 147 (65.9) | 0.031 | | 0.017 | |
| on insulin therapy | 260 (21.6) ​ | 155 (20.5) | 54 (23.8) | 51 (22.9) | 0.486 | | 0.336 | |
| eGFR, mL/min/1.73 m², (SD) | 56.6 (29.0) | 58.4 (28.8) | 57.1 (26.4) | 49.8 (31.1) | <0.001 | | 0.003 | |
| eGFR <45, without dialysis* | 211 (17.5) | 123 (16.3) | 46 (20.3) | 42 (18.8) | 0.313 | | 0.241 | |
| Dialysis* | 123 (10.2) | 60 (7.9) | 20 (8.8) | 43 (19.3) | <0.001 | | <0.001 | |
| Previous stroke* | 200 (16.6) ​ | 122 (16.2) | 33 (14.5) | 45 (20.2) | 0.247 | | 0.265 | |
| Atrial fibrillation* | 64 (5.3) ​ | 42 (5.6) | 10 (4.4) | 12 (5.4) | 0.838 | | 0.775 | |
| Old myocardial infarction* | 492 (40.8) ​ | 223 (29.5) | 139 (61.2) | 130 (58.3) | <0.001 | | <0.001 | |
| Peripheral vascular disease* | 219 (18.2) ​ | 134 (17.7) | 40 (17.6) | 45 (20.2) | 0.682 | | 0.465 | |
| Chronic obstructive pulmonary disease* | 57 (4.7) ​ | 37 (4.9) | 9 (4.0) | 11 (4.9) | 0.832 | | 0.885 | |
| Anemia (hemoglobin <11.0 g/dL)* | 245 (20.3) ​ | 141 (18.7) | 53 (23.3) | 51 (22.9) | 0.179 | | 0.098 | |
| Thrombocytopenia (platelets <100 × 109 /L)* | 24 (2.0) ​ | 21 (2.8) | 1 (0.4) | 2 (0.9) | 0.037 | | 0.027 | |
| LVEF, (SD) | 54.1 (12.4) | 54.6 (12.7) | 53.3 (11.9) | 53.2 (11.5) | 0.165 | | 0.024 | |
| ≤40%* | 180 (14.9) | 115 (15.2) | 36 (15.9) | 29 (13.0) | 0.651 | | 0.502 | |
| Bare metal stent | 302 (25.1) ​ | 0 | 158 (69.6) | 144 (64.6) | <0.001 | | <0.001 | |
| First-generation drug-eluting stent | 111 (9.2) | 0 | 31 (13.7) | 80 (35.9) | <0.001 | | <0.001 | |
| Second-generation and newer drug-eluting stents | 96 (8.0) ​ | 0 | 43 (18.9) | 53 (23.8) | <0.001 | | <0.001 | |
| Drug-coated balloon | 18 (1.5) ​ | 0 | 3 (1.3) | 15 (6.7) | <0.001 | | <0.001 | |
| **(B) Surgical characteristics** |  |  |  |  |  | |  | |
| Off-pump* | 1056 (87.6) ​ | 668 (88.5) | 196 (86.3) | 192 (86.1) | 0.515 ​ | | 0.276 | |
| On-pump beating | 99 (8.2) ​ | 61 (8.1) | 19 (8.4) | 19 (8.5) | 0.974 | | 0.820 | |
| On-pump arrest | 50 (4.1) ​ | 26 (3.4) ​ | 12 (5.3) ​ | 12 (5.4) ​ | 0.282 | | 0.137 | |
| Operation time | 338 (286–394) | 336 (284–390) | 338 (289–410) | 343 (290– 390) | 0.421 | | 0.227 | |
| Number of anastomoses, (SD) | 3.30 (0.95) | 3.31 (0.96) | 3.31 (0.99) | 3.23 (0.85) | 0.496 | | 0.313 | |
| Number of arterial anastomoses, (SD) | 2.23 (0.98) | 2.26 (0.96) | 2.26 (1.04) | 2.09 (0.99) | 0.072 | | 0.046 | |
| Number of venous anastomoses, (SD) | 1.06 (0.93) | 1.04 (0.93) | 1.04 (0.98) | 1.12 (0.91) | 0.536 | | 0.402 | |
| Number of arterial grafts, (SD) | 1.95 (0.70) | 1.96 (0.68) | 1.96 (0.74) | 1.83 (0.70) | 0.019 | | 0.046 | |
| Internal thoracic artery use* | 1195 (99.2) ​ | 752 (99.6) | 222 (97.8) | 221 (99.1) | 0.031 | | 0.167 | |
| Target of chronic total occlusion* | 417 (34.6) ​ | 288 (38.1) | 67 (29.5) | 62 (27.8) | 0.003 | | 0.001 | |
| Endarterectomy* | 27 (2.2) ​ | 14 (1.9) | 8 (3.5) | 5 (2.2) | 0.329 | | 0.469 | |
| Intraoperative blood transfusion | 588 (48.8) ​ | 349 (46.2) | 111 (48.9) | 128 (57.4) | 0.014 | | 0.005 | |
| Total arterial revascularization | 400 (33.2) | 255 (33.8) | 79 (34.8) | 66 (29.6) | 0.443 | | 0.334 | |
| **(C) Medication at discharge** |  |  |  |  |  | |  | |
| Antiplatelet therapy | 1181 (98.0) | 744 (98.5) | 222 (97.8) | 215 (96.4) | 0.131 | | 0.046 | |
| Dual antiplatelet therapy | 392 (32.5) ​ | 230 (30.5) | 78 (34.4) | 84 (37.7) | 0.106 | | 0.034 | |
| Aspirin* | 1133 (94.0) | 721 (95.5) | 214 (94.3) | 198 (88.8) | 0.001 | | <0.001 | |
| Clopidogrel* | 282 (23.4) ​ | 163 (21.6) | 52 (22.9) | 67 (30.0) | 0.032 | | 0.014 | |
| Cilostazol | 42 (3.5) ​ | 21 (2.8) | 10 (4.4) | 11 (4.9) | 0.215 | | 0.088 | |
| Ticagrelor | 2 (0.2) ​ | 1 (0.1) | 1 (0.4) | 0 (0.0) | 0.483 | | 0.916 | |
| Dipyridamole | 18 (1.5) ​ | 8 (1.1) | 3 (1.3) | 7 (3.1) | 0.077 | | 0.036 | |
| Prasugrel | 2 (0.2) ​ | 0​ | 0​ | 2 (0.9) | 0.012 | | 0.009 | |
| Ticlopidine | 187 (15.5) ​ | 113 (15.0) | 41 (18.1) | 33 (14.8) | 0.501 | | 0.795 | |
| Warfarin | 490 (40.7) ​ | 296 (39.2) | 93 (41.0) | 101 (45.3) | 0.265 | | 0.111 | |
| DOAC​ | 13 (1.1) ​ | 9 (1.2) | 3 (1.3) | 1 (0.4) | 0.593 | | 0.422 | |
| Statins* | 564 (46.8) ​ | 354 (46.9) | 100 (44.1) | 110 (49.3) | 0.532 | | 0.713 | |
| Beta-blockers* | 499 (41.4) ​ | 323 (42.8) | 82 (36.1) | 94 (42.2) | 0.197 | | 0.517 | |
| ACE-I/ARB* | 477 (39.6) ​ | 294 (38.9) | 102 (44.9) | 81 (36.3) | 0.147 | | 0.857 | |
| ARNI | 4 (0.3) ​ | 4 (0.5) | 0 (0.0) | 0 (0.0) | 0.302 | | 0.154 | |
| SGLT2 | 48 (4.0) ​ | 37 (4.9) | 4 (1.8) | 7 (3.1) | 0.082 | | 0.099 | |

* Risk-adjusted variables selected for Cox proportional hazards models.

Continuous variables are expressed as mean ± standard deviation (SD) or median (interquartile range). Categorical variables are expressed as numbers (percentages). Values were missing for operation time in one patient. eGFR, estimated glomerular filtration rate; LVEF, left ventricular ejection fraction; DOAC, direct oral anticoagulant; ACE-I, angiotensin-converting enzyme inhibitor; ARB, angiotensin II receptor blocker; ARNI, angiotensin receptor neprilysin inhibitor; SGLT2: sodium-glucose cotransporter 2

**Supplementary Table 2: Distribution of prior PCI by coronary territory**

| Coronary Territory | No PCI (n=755) | Single-lesion PCI (n=227) | Multiple-lesion PCI (n=223) | P-value |
| --- | --- | --- | --- | --- |
| LAD | 0 | 72 (31.7%) | 191 (85.7%) | <0.001 |
| LMT | 0 | 0 | 51 (22.9%) | <0.001 |
| RCA | 0 | 141 (62.1%) | 212 (95.1%) | <0.001 |
| LCx | 0 | 24 (10.6%) | 148 (66.4%) | <0.001 |

LAD, left anterior descending artery; LMT, left main trunk; RCA, right coronary artery; LCx, left circumflex artery

Patients in the multiple-lesion PCI group have stents in ≥2 territories by definition. LMT stenting was classified as two-territory involvement per study protocol.

**Supplementary Table 3: Early graft patency within 30 days postoperatively**

| Conduit type | Total grafts assessed | Occluded grafts | Patency rate (%) |
| --- | --- | --- | --- |
| Arterial grafts |  |  |  |
| LITA | 1,100 | 20 | 98.2 |
| RITA | 620 | 16 | 97.4 |
| RA | 412 | 5 | 98.8 |
| RGEA | 115 | 4 | 96.5 |
| Venous grafts |  |  |  |
| SVG | 863 | 51 | 94.1 |
| Overall | 3,110 | 96 | 96.9 |

Early graft patency was assessed in 1,140 of 1,205 patients (94.6%) who underwent coronary angiography or computed tomography angiography within 30 days postoperatively.

LITA, left internal thoracic artery; RITA, right internal thoracic artery; RA, radial artery; RGEA, right gastroepiploic artery; SVG, saphenous vein graft

**Supplementary Table 4: Angiographic findings of myocardial infarction cases (n = 24)**

| Characteristics | n (%) |
| --- | --- |
| Time from CABG to MI, years [median (IQR)] | 4.77 [2.8–11.7] |
|  |  |
| Culprit lesion location |  |
| LAD territory | 3 (12.5) |
| LCx territory | 6 (25.0) |
| RCA territory | 15 (50.0) |
| Previously stented lesion | 10 (41.7) |
| Non-bypassed territory | 5 (20.8) |
|  |  |
| Details of non-bypassed territory |  |
| Previously stented lesion | 2 (8.3) |
| Native vessel without prior stent | 3 (12.5) |
|  |  |
| Status of bypass graft to culprit territory |  |
| Bypassed territory | 19 (79.2) |
| Conduit type (n = 19) |  |
| LITA | 2 (10.5) |
| RITA | 1 (5.3) |
| RA | 1 (5.3) |
| RGEA | 2 (10.5) |
| Conventional SVG | 13 (68.4) |
|  |  |
| Graft status (n = 19) |  |
| Patent | 2 (10.5) |
| Occluded/Stenosed | 17 (89.5) |
|  |  |
| Treatment strategy |  |
| PCI | 18 (75.0) |
| Re-CABG | 0 |
| Medical therapy | 6 (25.0) |
|  |  |
| In-hospital mortality | 9 (37.5) |

LAD, left anterior descending artery; LCx, left circumflex artery; RCA, right coronary artery; LITA, left internal thoracic artery; RITA, right internal thoracic artery; RA, radial artery; RGEA, right gastroepiploic artery; SVG, saphenous vein graft

**Supplemental Figure Legends**

**Supplemental Figure 1.** Presents a stacked bar graph illustrating the annual percentage distribution of graft types used in coronary artery bypass grafting (CABG) from 2000 to 2024. Graft types include the left internal thoracic artery (LITA, red), right internal thoracic artery (RITA, yellow), radial artery (RA, blue), right gastroepiploic artery (RGEA, purple), conventional saphenous vein graft (C-SVG, gray), and no-touch saphenous vein graft (NT-SVG, green). The graph depicts evolving graft selection strategies and yearly variations in the use of each conduit.

**Supplemental Figure 2.** Displays annual trends in CABG cases according to prior percutaneous coronary intervention (PCI) status. Blue bars represent patients without prior PCI, red bars indicate those with prior PCI, and the green line illustrates the yearly proportion of patients with a history of PCI.
